# *Coxiella burnetii* in Bulk Tank Milk Samples, United States

**DOI:** 10.3201/eid1104.041036

**Published:** 2005-04

**Authors:** Sung Guk Kim, Eun Hee Kim, Caroline J. Lafferty, Edward Dubovi

**Affiliations:** * Cornell University, Ithaca, New York, USA

**Keywords:** dispatch, Trans-PCR, Q fever, Coxiella burnetii, bulk tank milk, insertion sequence, transposon

## Abstract

Dairy cattle are a primary reservoir of *Coxiella burnetii*, which causes Q fever. However, no recent nationwide studies have assessed the prevalence and risks of Q fever in dairy cattle. We report ≥94% prevalence in samples of bulk tank milk from U.S. dairy herds tested during the past 3 years.

Q fever is a ubiquitous zoonosis caused by *Coxiella burnetii*, an obligate intracellular rickettsial organism. Since the first independent reports by Australian and American investigators in 1935, Q fever has been found throughout the world, except New Zealand (*1*). *C. burnetii* infections have been reported in humans, farm animals, pet animals, wild animals, and arthropods (*2*). Among farm animals, dairy cattle, sheep, and goats are the major reservoirs of *C. burnetii*. Animals are often naturally infected but usually do not show typical symptoms of *C. burnetii* infection. Clinical signs of *C. burnetii* infection are abortion in sheep and goats and reproductive disorders in cattle (*1**,**3*). *C. burnetii* can be isolated from the blood, lungs, spleen, and liver of infected animals in the acute phase of the disease. The uterus and mammary glands are primary sites of infection in the chronic phase of *C. burnetii*. Shedding of *C. burnetii* into the environment occurs mainly during parturition by birth products, particularly the placenta of sheep. Also, shedding of *C. burnetii* in milk by infected dairy cattle is well documented (*1**,**3*).

Previous studies on the prevalence of *C. burnetii* in dairy cattle were based mainly on serologic tests, including complement fixation, indirect immunofluorescent assay (IFA), and enzyme-linked immunosorbent assay (ELISA). However, the seroprevalence of *C. burnetii* infection in cattle varies widely from 1 country to another and from 1 state to another in the United States. In Japan, a prevalence of 1.1% to 3.9% of *C. burnetii* infection in cattle was reported in the 1950s. However, a 1992 survey reported that 29.5% of healthy cattle and 84.3% of cattle with reproductive disorders in Japan had antibodies to *C. burnetii* shown by using IFA (*4*). In Canada, 67% of the 200 dairy herds were ELISA-positive for antibodies to *C. burnetii* (*5*). The reported seroprevalence of Q fever in the United States varies from 1% to 73%. Reports from the same state show wide differences depending on testing methods and the year of surveys; for example, the seroprevalence in Wisconsin was 33% in 1957 but 73% in 1962 (*6*). Seroepidemiologic studies have indicated that *C. burnetii* antibody seroprevalence in cattle has increased from the prevalence 20 or 30 years ago (*7*). However, the real prevalence of *C. burnetii* infection in cattle is not available, due in part to the lack of surveillance (*8*). Shedding of *C. burnetii* in milk by infected cattle was shown in studies conducted during the 1940s and 1950s.

Isolation of the Q fever agent by laboratory workers is difficult because the agent has a high infectivity rate, it is cumbersome in in vitro culture conditions, and handling it requires rigorous compliance requirements. Q fever is considered a "select agent" because it can potentially be used in bioterrorism and its handling is federally regulated. Recently, polymerase chain reaction (PCR) assays have been used to detect *C. burnetii* (*9*). A trans-PCR assay was implemented to detect *C. burnetii* in milk by targeting a transposon-like sequence found only in *C. burnetii* (*10*). The trans-PCR assay detects *C. burnetii* in samples immediately, unlike serologic assays that detect antibodies that could have been introduced months earlier.

A real-time PCR assay targeting IS*1111* was developed in this study to measure amounts of *C. burnetii* shed in milk. Our study was to assess the prevalence of *C. burnetii* in bulk milk samples from dairy herds in the United States by using PCR.

## The Study

The samples in this study were somatic cells extracted from bulk tank milk aliquots submitted to the New York State Animal Health Diagnostic Laboratory to detect bovine viral diarrhea that persistently infected lactating dairy cattle. The samples tested do not represent a random sampling, as tests were done only on samples available. The samples are heavily weighted to the Northeast, but some are from the Midwest and West. We tested 316 bulk tank milk samples from dairy herds in the United States during a 3-year period from January 2001 to December 2003 by using trans-PCR (Figure). Positive results were confirmed by nested PCR and DNA sequencing. The sequencing results of the 687-bp PCR product were consistent with the published sequence of IS*1111* with 100% homology. A summary of the PCR test results of the bulk tank samples is shown in Table 1. The overall prevalence of *C. burnetii* in the tested samples was 94.3% with little variation (93.2% to 94.7%) from year to year. Samples from New York State did not show significant variation from other states, which indicates that *C. burnetii* infection in the dairy herds was persistent or steady, with little temporal or regional variations. Milk samples were collected 6 times from 2002 to 2004 from specific cattle in a small, *C. burnetii*–positive dairy to monitor the infection in specific cattle. A summary of the results of tests on milk samples from specific cattle is presented in Table 2. While 28 (52.8%) of 53 cattle were *C. burnetii* positive in 2002, 23.5% and 31.3% of the cattle were positive in 2003 and 2004, respectively. Daily and weekly shedding levels of 5 *C. burnetii*– positive cattle were assayed by real-time PCR. Real-time PCR was conducted by using the primers and probe designed by the Primer Express program (Applied Biosystems, Foster City, CA). The primer set consisted of primers trans-f (5´-GGGTAAAACGGTGGAACA ACA-3´) and trans-r (5´-ACAACCCCCGAATCTCATTG-3´). The internal probe trans-p (5´-AACGATCGCGTATCTTTAACAGCGCTTG-3´) was labeled with the reporter dye 5-carboxyfluoroscein (FAM) on the 5´ end and the quencher dye N´, N´, N´, N´-tetramethyl-6-carboxyrhodamine (TAMRA) at the 3´ end. The reactions and assay conditions were according to the manufacturer's instructions (Applied Biosystems). Each cattle shed a similar amount of *C. burnetii* daily over 7 days; weekly shedding over 4 weeks was also similar. Shedding levels of positive cattle were estimated to be 10^1^–10^4^ cells/mL each. The bulk tank milk samples of the herd stayed at a level of 10^2^ cells/mL over 3 years.

**Figure:**
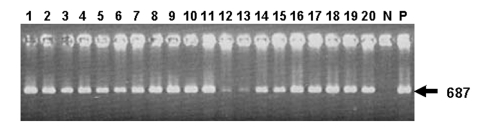
Agarose gel electrophoresis of *Coxiella burnetii* Trans-polymerase chain reaction products amplified from total DNA of bulk tank milk samples. Lanes 1 to 20, bulk tank milk samples; N, water negative control; P, positive control (DNA of Nine Mile strain). The arrow indicates the amplification of a 687-bp fragment in the IS*1111* sequence of *C. burnetii*.

**Table 1:** Prevalence of Q fever in bulk tank milk of U.S. dairy herds (2001–2003)

Year	State	No. of samples	Positive (%)	Negative (%)
2001	New York State	20	18 (90.0)	2 (10.0)
	Other states*	24	23 (95.8)	1 (4.2)
	Subtotal	44	41 (93.2)	3 (6.8)
2002	New York State	61	58 (95.1)	3 (4.9)
	Other states*	60	56 (93.3)	4 (6.7)
	Subtotal	121	114 (94.2)	7 (5.8)
2003	New York State	43	40 (93.0)	3 (7.0)
	Other states*	108	103 (95.4)	5 (4.6)
	Subtotal	151	143 (94.7)	8 (5.3)
	Total	316	298 (94.3)	18 (5.7)

*CA, CT, CO, IL, IN, MA, MD, MI, MN, MO, NC, OH, PA, UT, VA, VT, WA, WI.

**Table 2:** Q fever infection rate of specific cows in a bulk tank–positive dairy herd based on Coxiella burnetii shedding in their milk over 3 years (2002–2004)

Month/year collected	No. of samples	Positive (%)	Negative (%)
Jul 2002	53	28 (52.8)	25 (52.8)
Aug 2002	53	28 (52.8)	25 (52.8)
Sep 2003	51	16 (31.3)	35 (68.6)
Dec 2003	48	14 (29.2)	34 (70.8)
Feb 2004	52	13 (25.0)	39 (75.0)
Jul 2004	34	8 (23.5)	26 (76.5)

## Conclusions

Bulk tank milk has been used for surveillance samples in dairy herds for several bovine diseases including bovine viral diarrhea. More than 90% of U.S. dairy herds sampled were infected with *C. burnetii* based on bulk tank milk testing over a 3-year period. This high prevalence did not show temporal or regional variation, suggesting that *C. burnetii* infections in dairy herds are common throughout the United States. Our report of *C. burnetii* in dairy herds is not surprising if earlier reports regarding an increase of bovine infection in North America are considered. An early investigator concluded that *C. burnetii* was endemic throughout the United States in the 1950s and predicted that a high bovine infection rate could occur in other parts of the country, as it did in southern California where a 98% infection rate was reported (*11*). A California survey reported that 20 (100%) of 20 herds in 17 counties throughout the state contained seropositive cattle, and 82% of 1,052 specific cattle from the herds were seropositive (*12*). An increase in the prevalence of Q fever from 2.3% in 1964 to 66.8% in 1984 was reported in Ontario dairy herds (*13*). Our study found a notable decrease in shedding of *C. burnetii* in milk by specific cattle from 52.8% to 23.5% over 3 years (Table 2). This decrease was not because animals stopped shedding the organism but because uninfected animals replaced shedding animals with an average annual replacement rate of 30%. With the exception of the first year of our study, the shedding rate appeared to be steady at 20% to 30% over 2 years. A similar shedding rate was found in a California study; 23% of 840 cattle were shedding *C. burnetii* in their milk (*13*). Continual daily and weekly shedding in the milk by the infected cattle suggests chronic infection by *C. burnetii*. Chronic *C. burnetii* infection of dairy cattle could be the most important source of human infection simply based on sheer numbers (*1*). Extrapolation of our data to the national dairy herd suggests that nearly 3 million lactating cattle are shedding *C. burnetti* daily. Though the mode and extent of transmission from bovine to human has not been determined, epidemiologic studies indicate that Q fever develops in farmers, veterinarians, and slaughterhouse workers who are in contact with domestic animals (*14*). While infection from commercial milk is unlikely because of the pasteurization process, ingestion of raw milk has been linked to higher seroprevalence rates.

In the aftermath of September 11, 2001, and the anthrax incidents, the use of biologic warfare is no longer a distant possibility. *C. burnetii* is considered a potential bioterrorism agent because of its high infectivity (a single organism may cause disease in human), its ease of dispersion in aerosols (because of its small, sporelike structure), and its resistance to extreme environmental conditions and chemicals (*15*). Therefore, further investigations are needed to determine the implications of the high prevalence of *C. burnetii* in dairy herds, to address the potential risk to public health, and to be prepared for outbreaks and bioterrorism events. Currently, no commercial vaccines are available for cattle, and no effective treatment protocol exists for infected animals.
